# Infliximab treatment for Cronkhite-Canada syndrome in pregnancy: A case report

**DOI:** 10.1016/j.crwh.2025.e00706

**Published:** 2025-03-30

**Authors:** Ayano Rosemary Nakamura, Shuji Yamamoto, Yoshitsugu Chigusa, Masaki Mandai, Haruta Mogami

**Affiliations:** a Department of Gynecology and Obstetrics, Graduate School of Medicine, Kyoto University, 54 Shogoin Kawahara-cho, Sakyo-ku, Kyoto 606-8507, Japan; b Department of Gastroenterology and Hepatology, Graduate School of Medicine, Kyoto University, 54 Shogoin Kawahara-cho, Sakyo-ku, Kyoto 606-8507, Japan

**Keywords:** Anti-TNFα therapy, Cronkhite-Canada syndrome, Hypoalbuminemia, Infliximab, Pregnancy

## Abstract

Cronkhite-Canada syndrome (CCS) is a rare nonhereditary disorder characterized by gastrointestinal polyps and protein-losing enteropathy. While an increasing number of CCS cases have been reported worldwide, no documented cases involving pregnant patients could be found. Consequently, optimal management strategies for CCS during the preconception period and pregnancy remain unclear., The present report concerns the case of a 36-year-old woman with steroid-refractory CCS stabilized with gastrointestinal surgeries and infliximab, an anti-tumor necrosis factor-α (TNF-α) agent, who became pregnant. Infliximab was continued throughout pregnancy and postpartum. Despite persistent hypoalbuminemia, sh delivered a healthy infant weighing 2518 g vaginally at 38 weeks and 2 days without CCS exacerbation. Both the patient and her infant experienced an uneventful postpartum course. This case suggests that maintaining disease control with anti-TNF-α therapy in pregnant patients with CCS may contribute to optimizing maternal and neonatal outcomes.

## Highlights

• First documented case of pregnancy in a patient with Cronkhite-Canada syndrome (CCS).
• Infliximab maintained CCS disease control from periconception through postpartum period.
• Anti-TNFα therapy may contribute to favorable maternal and neonatal outcomes in CCS.

## 1 Introduction

Cronkhite-Canada syndrome (CCS) is a rare, nonhereditary disorder characterized by multiple non-neoplastic polyps in the stomach and colon, gastrointestinal symptoms such as diarrhea, and a distinctive dermatological triad of nail atrophy, skin hyperpigmentation, and hair loss [1,2]. The incidence of CCS is extremely low, estimated at 1 per 1,000,000 individuals, with a mean age at diagnosis of 63.6 years [2,3]. Polyps develop throughout the gastrointestinal tract, excluding the esophagus, leading to complications such as gastrointestinal bleeding with anemia, intussusception, hypoproteinemia, malnutrition, electrolyte imbalances, inflammatory bowel disease, and vitamin deficiencies [2,4]. Clinically significant concerns include protein-losing gastroenteropathy, which leads to malnutrition and hypogammaglobulinemia, predisposing patients to immunodeficiency. Additionally, CCS carries a significant risk of gastric and colorectal cancer, with malignancies reported in nearly 30 % of cases [4]. While the exact etiology remains unclear, immune dysregulation is strongly suspected to play a pivotal role. Consequently, treatment primarily involves immunosuppressive therapy, particularly corticosteroids. However, in some cases, pharmacological treatment alone is insufficient, necessitating surgical intervention or alternative therapies.

While an increasing number of CCS cases have been reported worldwide, no cases involving pregnant patients could be found in the literature. Consequently, optimal management strategies for CCS during the preconception period and pregnancy remain unclear. The case reported here (seemingly the first of CSS in pregnancy) was particularly challenging due to steroid resistance; however, the initiation of infliximab, an anti-tumor necrosis factor-α (TNF-α) antibody, prior to conception and its continuation throughout pregnancy and postpartum resulted in a favorable outcome.

## 2 Case Presentation

**Fig. 1:**
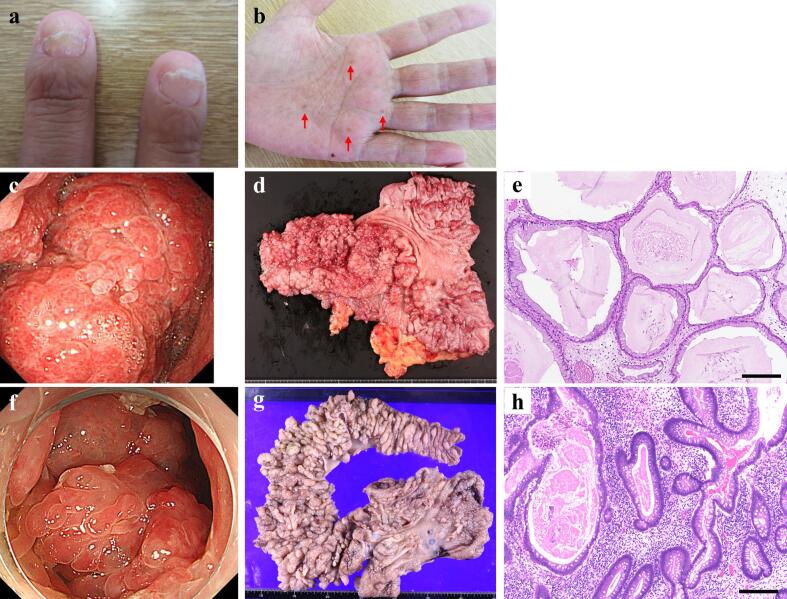
Diagnostic features of Cronkhite-Canada syndrome. (a) Characteristic nail dystrophy showing atrophy and onycholysis. (b) Skin hyperpigmentation on the patient's hand (arrows). (c, f) Endoscopic images showing multiple polyps in the stomach and colon, respectively. (d, g) Macroscopic findings of the resected specimens from distal gastrectomy and partial colectomy, showing numerous polyps. (e, h) Microscopic findings of polyps in stomach and colon showing glandular structures with variable dilatation, accompanied by edematous changes in the stroma and inflammatory cell infiltration. Hematoxylin and eosin stain, magnification ×100, scale bars = 200 μm.

**Fig. 2:**
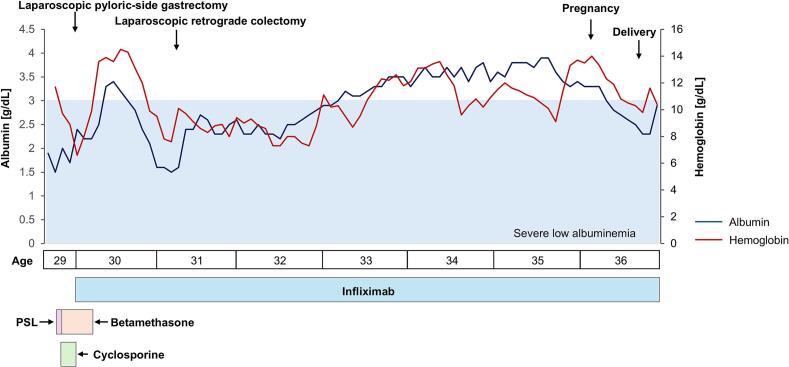
Treatment course and changes in hemoglobin and serum albumin levels. PSL: prednisolone.

A 36-year-old multiparous woman (gravida 3, para 1) presented to the hospital obstetrics department at 10 weeks of gestation. She had an approximately eight-year history of CCS. At age 28, she had noticed nail atrophy, palmar pigmentation (Fig. 1a, b), and postprandial epigastric pain. She had given birth to her first child at the age of 29 and subsequently began experiencing frequent diarrhea. Upper and lower gastrointestinal endoscopy revealed numerous polyps in the stomach and colon. Based on these clinical findings and severe hypoalbuminemia, the patient was diagnosed with CCS. Treatment was initiated with prednisolone (PSL) at 35 mg/day (0.6 mg/kg) and total parenteral nutrition (Fig. 2). However, due to persistent symptoms, PSL was increased to 60 mg/day (1.0 mg/kg), and cyclosporin (2.5 mg/kg) was initiated. Despite these interventions, her symptoms did not improve, and electrolyte imbalances, including hypocalcemia, hypomagnesemia, and hypophosphatemia due to intestinal leakage, persisted. PSL was subsequently shifted to betamethasone due to suspected anaphylaxis.

The patient was then referred to the hospital gastroenterology department. Enlarged gastric polyps were found to be causing pyloric obstruction (Fig. 1c), vomiting, and gastrointestinal bleeding, necessitating the discontinuation of cyclosporine. Given the failure of conventional therapies, treatment with infliximab was considered, as disease control with other medications was extremely challenging in this case. The off-label use of this medication was managed according to institutional guidelines. The risks and benefits of infliximab therapy were thoroughly explained to the patient, and treatment was initiated after obtaining informed consent (5 mg/kg at 0, 2, and 6 weeks as an induction dose, followed by 5 mg/kg every 8 weeks for maintenance). Moreover, laparoscopic distal gastrectomy with Roux-en-Y reconstruction was performed (Fig. 1d, e), resulting in the improvement of gastrointestinal symptoms.

**Fig. 3:**
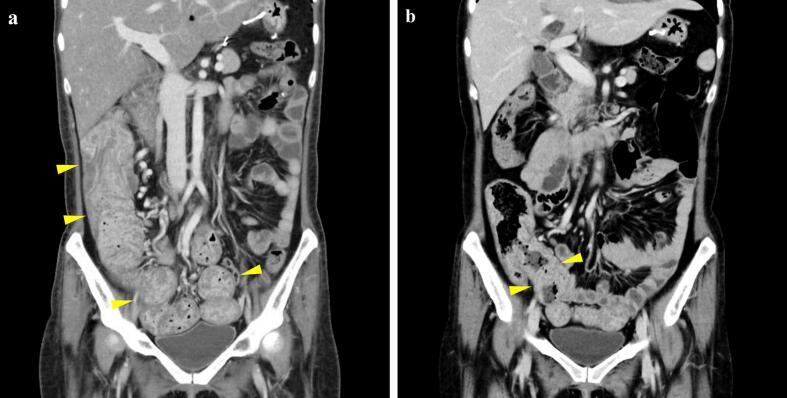
Abdominal computed tomography images (a) 21 days and (b) 121 days after initiation of infliximab. Multiple mass shadows suspected to be ileal and colonic polyps (arrowheads) show reduction in size.

At age 30, exacerbation of dermatological symptoms and a rapid decline in serum albumin levels necessitated an increase in the dose of infliximab to 500 mg (10 mg/kg every 8 weeks). At age 31, as the severe hypoalbuminemia had persisted, lower gastrointestinal endoscopy was performed; it revealed polyp enlargement in the colon (Fig. 1f). Consequently, laparoscopic partial colectomy and distal ileal resection were performed (Fig. 1g, h). According to the histological examination of the resected stomach and colon specimens, the lesions demonstrated glandular structures with variable dilatation, accompanied by edematous changes in the stroma and inflammatory cell infiltration. These histopathological findings are consistent with CCS. Following surgery, disease activity remained well controlled with infliximab monotherapy at 500 mg (10 mg/kg every 4–5 weeks) for nearly five years. Computed tomography images taken 14 days and 121 days after the initiation of infliximab (and after the gastrectomy – see Fig. 2), revealed a reduction in the volume of multiple ileal colonic polyps, suggesting the effectiveness of infliximab therapy (Fig. 3).

Though the patient had desired pregnancy, she had initially prioritized treatment due to the severity of her CCS. At age 35, after achieving disease stabilization with infliximab, she expressed a strong desire to conceive. The patient and her family were counseled that no data existed regarding how pregnancy might affect CCS, and were warned that potential disease exacerbation could be life-threatening. They were also informed about the safety profile of infliximab during pregnancy and lactation, and the need to continue this therapy from preconception through postpartum period. After comprehensive counseling, the patient was advised to attempt conception within approximately one year, provided her CCS remained stable.

**Fig. 4:**
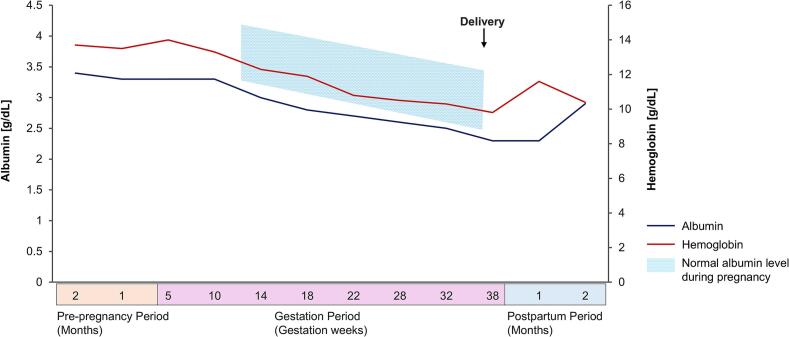
Changes in hemoglobin and serum albumin levels during pregnancy and the peripartum period.

At age 36, she conceived and prenatal hospital care was initiated. Prior to pregnancy, the patient had been receiving iron supplementation for anemia, which was continued throughout gestation. At 23 weeks of gestation, her serum zinc level decreased to 46 μg/dL, prompting the initiation of zinc supplementation. Before pregnancy, the serum albumin level had never fallen below 3 g/dL; however, it decreased to 2.8 g/dL at 18 weeks of gestation and to 2.3 g/dL at 38 weeks of gestation. Despite this, hypoalbuminemia remained stable without further deterioration (Fig. 4). The patient delivered an infant weighing 2518 g at 38 weeks and 2 days of gestation via vaginal delivery. The infant had Apgar scores of 8 and 9, and umbilical arterial blood pH of 7.146. Infliximab monotherapy at 500 mg (10 mg/kg every 4 weeks), continued throughout pregnancy and postpartum, maintained disease control.

## 3 Discussion

This case report is the first, to the authors' knowledge, to provide an account of pregnancy and delivery in a woman with CCS. Since CCS was first described in 1955 [5], only slightly over 500 cases have been reported worldwide, highlighting its rarity [6,7]. Moreover, approximately 80 % of CCS cases are diagnosed in individuals over the age of 50, with a male-to-female ratio of 3:2 [6], suggesting that CCS is exceedingly rare in women of reproductive age. However, with global trends toward delayed marriage and childbirth, cases of pregnancy complicated by CCS, although still uncommon, warrant clinical attention. This report provides valuable insights into the management of pregnancy in women with CCS and may serve as an important reference for future clinical practice.

A notable feature of this case is that infliximab therapy was initiated before pregnancy and continued uninterrupted throughout pregnancy and the postpartum period. While corticosteroids remain the cornerstone of CCS treatment, disease control was not achieved with steroid therapy alone in this patient. Given the severity of the disease, which necessitated surgical intervention, a biologic agent was introduced as an alternative treatment [1]. Although the pathogenesis of CCS remains unclear, increased TNF-α expression in the intestinal mucosa [8] and mutations in the protein kinase, DNA-activated, catalytic subunit (*PRKDC*) gene—implicated in the regulation of inflammatory cytokines, including TNF-α [9]—suggest a potential therapeutic role for anti-TNF-α antibodies. Indeed, several reports have documented remission in steroid-refractory CCS cases following infliximab therapy [[10], [11], [12], [13]]. In the present case, the initiation and dose escalation of infliximab appeared to stabilize disease activity, as evidenced by the absence of gastrointestinal polyposis progression and improved serum albumin and hemoglobin levels before conception.

During pregnancy, serum albumin and hemoglobin levels typically decline as gestation progresses. A similar pattern was observed in this case, with serum albumin levels falling below the normal pregnancy range (Fig. 2). Maternal serum albumin levels during pregnancy are known to be associated with fetal growth and the risk of preterm delivery [14]. However, in this case, the infant was born at full term weighing 2518 g (15.9 percentile), which did not meet the criteria for small for gestational age. This suggests that preventing hypoalbuminemia due to CCS exacerbation is a key consideration in managing pregnancies complicated by CCS. Furthermore, there has been a reported case in which CCS symptoms first became apparent postpartum, leading to a delayed diagnosis [15]. This highlights the potential importance of continuous infliximab therapy, not only before conception but also throughout pregnancy and the postpartum period, to maintain disease control and achieve favorable maternal and neonatal outcomes in pregnancies complicated by CCS.

The safety of anti-TNF-α agents during pregnancy has been well documented, primarily based on evidence from their use in pregnancies complicated by inflammatory bowel disease. Studies suggest that these agents are not associated with teratogenic effects or an increased risk of fetal infections [16,17]. Nevertheless, as IgG transport across the placenta begins in the second trimester and becomes more active in the third trimester [18], the transfer of anti-TNF-α antibodies to the fetus must be considered if therapy is continues into late pregnancy. Given this transfer, live vaccines should not be administered to the infant for the first six months after birth. Indeed, a case has been reported in which a neonate died shortly after birth following bacillus Calmette-Guérin vaccination, raising concerns about the timing of live vaccinations in infants exposed to anti-TNF-α agents in utero. Regarding lactation, due to the large molecular size of anti-TNF-α antibodies, their transfer into breast milk is believed to be minimal [16]. Consequently, breastfeeding while receiving anti-TNF-α therapy is generally considered safe.

In this case, in addition to infliximab therapy, surgical interventions may have contributed to disease improvement. Several case reports have documented favorable outcomes with surgical interventions in CCS patients [19,20]. In the case reported here, rapid enlargement of gastric polyps led to pyloric obstruction, resulting in frequent vomiting and intolerable distress, necessitating laparoscopic distal gastrectomy for symptomatic relief. Subsequently, enlargement of colonic polyps caused worsening of protein-losing gastroenteropathy that was refractory to pharmacological treatment, prompting laparoscopic partial colectomy. Following these surgical interventions, the patient's condition remained stable for an extended period with continued infliximab administration. While careful case selection is necessary, surgical therapy may be considered a valuable treatment option for female CCS patients desiring pregnancy, as disease stabilization prior to conception is essential for optimal outcomes.

## 4 Conclusion

This case suggests that continuous infliximab therapy, initiated before conception and maintained throughout pregnancy and postpartum, may have contributed to disease control of steroid-refractory CCS, potentially helping to achieve favorable maternal and neonatal outcomes. While further case reports are required to establish optimal management strategies for pregnancies complicated by CCS, achieving stable disease control prior to conception is undoubtedly critical. When used with appropriate monitoring, anti-TNF-α agents can be safely administered during pregnancy and lactation, and treatment should not be discontinued solely due to pregnancy. This case report provides valuable clinical insights for preconception counseling and management strategies for women with CCS and their partners.
